# Twenty-four-hour ambulatory blood pressure variability and association with ischemic stroke subtypes in the subacute stage

**DOI:** 10.3389/fneur.2023.1139816

**Published:** 2023-04-17

**Authors:** Lijuan Wang, Xiaoshuang Xia, Xin Liu, Guilin Wu, Yanna Wang, Dongliang Yang, Peilin Liu, Zhuangzhuang Chen, Lin Wang, Xin Li

**Affiliations:** ^1^Department of Neurology, The Second Hospital of Tianjin Medical University, Tianjin, China; ^2^Department of Neurology, Beijing Zhongguancun Hospital, Beijing, China; ^3^Beijing Municipal Medical Insurance Bureau, Beijing, China; ^4^Department of Computer Teaching and Research Section, Cangzhou Medical College, Hebei, China; ^5^Department of Geriatrics, The Second Hospital of Tianjin Medical University, Tianjin, China

**Keywords:** ambulatory blood pressure variability, ischemic stroke, large-artery atherosclerosis, branch atheromatous disease, small-vessel disease, cardioembolic stroke

## Abstract

**Background and purpose:**

Blood pressure (BP) variability (BPV) increases the risk of cerebral disease in both hemorrhagic and ischemic strokes. However, whether BPV is associated with different types of ischemic stroke remains unclear. In this study, we explored the relationship between BPV and ischemic stroke subtypes.

**Methods:**

We enrolled consecutive patients aged 47–95 years with ischemic stroke in the subacute stage. We categorized them into four groups based on their artery atherosclerosis severity, brain magnetic resonance imaging markers, and disease history: large-artery atherosclerosis, branch atheromatous disease, small-vessel disease, and cardioembolic stroke. Twenty-four-hour ambulatory blood pressure monitoring was performed, and the mean systolic blood pressure/diastolic blood pressure, standard deviation, and coefficient of variation were calculated. A multiple logistic regression model and random forest were used to test the relationship between BP and BPV in the different types of ischemic stroke.

**Results:**

A total of 286 patients, including 150 men (73.0 ± 12.3 years) and 136 women (77.8 ± 9.6 years) were included in the study. Of these, 86 (30.1%) patients had large-artery atherosclerosis, 76 (26.6%) had branch atheromatous disease, 82 (28.7%) had small-vessel disease, and 42 (14.7%) had cardioembolic stroke. There were statistically significant differences in BPV between subtypes of ischemic stroke in 24-h ambulatory blood pressure monitoring. The random forest model showed that BP and BPV were important features associated with ischemic stroke. Multinomial logistic regression analysis demonstrated that systolic blood pressure levels; systolic blood pressure variability at 24 h, daytime and nighttime; and nighttime diastolic blood pressure were independent risk factors for large-artery atherosclerosis after adjustment for confounders. When compared to branch atheromatous disease and small-vessel disease, nighttime diastolic blood pressure and standard deviation of diastolic blood pressure were significantly associated with patients in the cardioembolic stroke group. However, a similar statistical difference was not seen in patients with large-artery atherosclerosis.

**Conclusion:**

The results of this study indicate a discrepancy in blood pressure variability among different ischemic stroke subtypes during the subacute stage. Higher systolic blood pressure and systolic blood pressure variability during the 24 h, daytime, and nighttime, and nighttime diastolic blood pressure were independent predictors for large-artery atherosclerosis stroke. Increased nighttime diastolic BPV was an independent risk factor for cardioembolic stroke.

## 1. Introduction

Hypertension is an important risk factor for cardiovascular and cerebrovascular disease (1, 2). Elevated blood pressure levels, particularly systolic blood pressure (SBP), significantly increase the risk of stroke, poor prognosis, and recurrence in patients with ischemic stroke (3, 4). Blood pressure variability (BPV), an increasing research interest in recent years, mainly reflects continuous fluctuations in blood pressure over hours, days, months, or years, including short-term, medium-term, and long-term blood pressure variability. Reports demonstrate that BPV, independent of the BP level, is strongly associated with heart disease, cognitive impairment, and stroke (5–7).

Twenty-hour ambulatory blood pressure monitoring (ABPM), which documents BP fluctuations during daytime and nighttime, is a more valuable and scientific method for predicting pressure-related brain damage compared to a single blood pressure measurement (8). Previous studies have indicated that BPV positively correlates with ischemic stroke in both large- and small-vessel diseases. For example, a study found that ambulatory blood pressure variability (ABPV) was positively associated with carotid intima-media thickness and prompted the formation of atherosclerotic plaque in hypertensive patients. This suggested that BPV is the strongest predictor of extracranial large-artery disease (9). Meanwhile, higher BPV is linked with early intra-arterial thrombectomy treatment and adverse outcomes in patients with acute ischemic stroke with intracranial large-artery disease (10). Moreover, cerebral small-vessel disease (CSVD) is a common and potentially devastating condition in the elderly. Recent evidence indicated that 24-h ABPV is closely associated with the total CSVD burden as well as with different magnetic resonance imaging (MRI) markers of CSVD, such as white matter hyperintensity (WMH), enlarged perivascular spaces, and lacunar infarction. Together, they gradually aggravate the progression of CSVD (11–14). Simultaneously, nighttime BPV has been confirmed to increase the risk of cardioembolic stroke (CES), which is attributed to the increased risk of cardiovascular disease (15).

These results demonstrate that BPV and ischemic stroke are closely associated and are based on the differences in vascular anatomy, structure, and pathological changes between large and small intracranial vessels in the brain. We hypothesized that there may be differences in clinical characteristics and BPV variability among subtypes of ischemic stroke. However, these differences are poorly understood. Therefore, our novel study investigates the association between BPV and ischemic stroke subtypes. We aim to discover the different effects of BPV across subtypes of ischemic stroke and provide valuable implications for the targeted management of BP in patients with ischemic stroke.

## 2. Materials and methods

### 2.1. Ethics approval

This study was approved by the local ethics committee of Zhongguancun Hospital, Beijing, China (20230104). Due to the retrospective and observational design of this study, which posed no potential harm to the enrolled patients, the requirement for informed consent was waived. All medical records and personal information were anonymized and de-identified.

### 2.2. Study subject

This is a retrospective study that enrolled a consecutive series of patients with acute ischemic stroke within 24 h of admission to the Neurology Department of Beijing Zhongguancun Hospital Affiliated with the Chinese Academy of Sciences between 1 January 2019 and 30 April 2022. Ischemic stroke was defined as the sudden onset of neurologic dysfunction and associated new infarcts on cranial CT or brain MRI (16). All patients included in this study met the following inclusion criteria: (1) Those who were older than 18 years; (2) Had undergone 24-h ambulatory blood pressure monitoring (ABPM) in a subacute stage, defined as the period from 72 h after the onset of symptoms of ischemic stroke until the day of discharge or transfer to a rehabilitation unit (4–21 days) during which the patients were neurologically and medically stable (17); and (3) Had undergone brain magnetic resonance imaging (MRI) and magnetic resonance angiogram (MRA) or computed tomography angiography (CTA). The exclusion criteria were as follows: (1) hospitalization stay of <72 h; (2) Hemorrhagic stroke; and (3) Infarction caused by hematological system diseases, hereditary diseases, artery dissection, intracranial tumor, inflammation, or traumatic brain injury. The early management of ischemic stroke was in accordance with the 2018 American Heart Association/American Stroke of Anesthesiologists (AHA/ASA) guideline criteria (17).

### 2.3. Clinical information

Clinical data included demographics and stroke risk factors, such as age, sex, hypertension, diabetes mellitus (DM), dyslipidemia, coronary heart disease (CHD), atrial fibrillation, drinking, smoking, and National Institutes of Health Stroke Scale (NIHSS) scores on admission. Laboratory tests included blood urea nitrogen (BUN), creatinine (Cr), fasting glucose (FGB), serum total cholesterol (TC), triglyceride (TG), low-density lipoprotein cholesterol (LDL-C), high-density lipoprotein cholesterol (HDL-C), homocysteine (HCY), hemoglobin, and C-reactive protein (CRP). Antihypertensive drugs used were classified as angiotensin-receptor blockers (ARB), angiotensin-converting enzyme inhibitors (ACEI), calcium-channel blockers (CCB), β-blockers, and diuretics.

### 2.4. Brain MRI analysis and etiological classification of ischemic stroke

All the patients included in this study underwent brain MRI using a 1.5-Tesla scanner (SIGNA Explore, Erlangen, Germany). The whole MRI sequence had a thickness of 5 mm both in the axial and sagittal planes. The parameters included were as follows: T1-weighted images (repetition time [TR]/echo time [TE] = 1750/29), T2-weighted images (TR/TE = 5485/126), fluid attenuation inversion recovery (FLAIR; TR/TE =8400/150), diffusion-weighted imaging (DWI; TR/TE = 4000/68), and susceptibility-weighted imaging (SWI; TR/TE = 75/47.4, slice thickness = 3mm). Three-dimensional time-of-flight (3D-TOF) magnetic resonance angiogram scans (TR/TE = 23/3.8, slice thickness = 1.4 mm) were obtained in the axial plane.

Vascular imaging, including MRA, CTA, or doppler ultrasonography, was used to assess the stenosis of the intracranial and extracranial arteries. The degree of stenosis was classified according to the North American Symptomatic Carotid Endarterectomy Trial (NASCET) criteria as follows: none, 0; mild, 0–49%; and moderate to severe, 50–99% (18, 19).

Acute infarction was defined based on DWI and the apparent diffusion coefficient (ADC) map. The maximum diameter of the infarcts was measured by DWI in axial planes, and the infarct location, size, and extent of vascular stenosis were evaluated by two professional neurologists (LijW and XLiu), who were blinded to the patient's characteristics. Discrepancies were resolved by consensus. The included patients were classified into four etiological subtypes according to the Trial of Org 10172 in Acute Stroke Treatment (TOAST) criteria and the definition of related-branch atheromatous (20–22): (1) Large-artery atherosclerosis (LAA): infarct lesions >15 mm in diameter and with significant (>50%) stenosis or occlusion in intracranial or extracranial arteries. (2) Branch atheromatous disease (BAD): infarct lesions in the lenticulostriate arterial (LAS) region with a diameter ≥15 mm, appeared in at least three consecutive axial slices (at a slice thickness of 5 mm) and in the absence of middle cerebral artery stenosis (>50%); or an infarct lesion in the pontine penetrating arterial (PPA) region that extends from the deep pons to the ventral pons unilaterally on the axial DWI in the absence basal artery stenosis (>50%) and potential sources of cardioembolic stroke. (3) Small-vessel disease (SVD): infarct lesion <15 mm in diameter, located in the white matter and the deep gray matter of the brain and brainstem and unaccompanied by ipsilateral large-artery stenosis (>50%). This category also includes infarct lesions in the LSA region with <3 slices or in the pontine region that does not extend to the ventral surface of the pons in order to distinguish them from BAD. (4) Cardioembolism (CE): the presence of at least one risk factor for the cardiac source of embolism (atrial fibrillation/acute myocardial infarction/mechanical prosthetic valve, etc.) and the absence of large-artery atherosclerosis.

### 2.5. 24-h ABPM and 24-h ABPV

Regular systolic and diastolic blood pressures were recorded using 24-h ABPM with a fully automatic oscillometric device (NC 27560, Sun Tech Medical. Morrisville, U.S.A, 2013). The device was validated according to the protocol of the British Hypertension Society (23). The BP was measured by well-trained research staff after the patient had rested for at least 5 min in a seated position. An appropriately sized BP cuff was fixed to the upper arm of non-hemiplegic patients with a stable clinical conditions. Patients were instructed to perform their usual activities and avoid excessive movement during the measurement. Measurements were taken every 30 min during the day (8 a.m. to 10:59 p.m.) and every 60 min during the night (11 p.m. to 7:59 a.m.). The definition of day and night is based on an automated system with standard time windows. The results were considered usable if more than 80% of the measurements were valid. The 24-h ABPV was represented by several indices: mean systolic blood pressure (SBP)/diastolic blood pressure (DBP), coefficient of variation (CV = SD / mean BP × 100 [%]) (24), and standard deviation (SD) of SBP and DBP in 24 h, daytime, and nighttime. In addition to 24-h ABPV, we calculated the nocturnal dip (%) (25), which represents the percentage of systolic blood pressure drop at night.


$$\begin{array}{l} \frac{\begin{array}{c} mean\;SBP_{daytime}-\;mean\;SBP_{nightime}\\ \; \end{array}}{mean\;SBP_{daytime}}\times 100 \end{array}$$

Dipping category: extreme dipper (> 20%), dipper (≥ 10% and ≤ 20%), non-dipper (0–10%), and reverse-dipper (< 0%). Patients continued to use antihypertensive medication during the study period.

### 2.6. Statistical analysis

Distributions of continuous variables are presented as mean (SD) for normally distributed variables and medians (interquartile range) for abnormally distributed variables. One-way ANOVA tests or non-parametric tests were used where appropriate. Categorical variables are presented as percentages and chi-squared tests were used. For multiple comparisons between groups, the Bonferroni test and Kruskal–Wallis H-test were used for normally distributed continuous and anomalous continuous variables, respectively. Multiple logistic regression analysis was performed to identify the association between ABPV and each ischemic stroke subtype. The dependent variable was the subtype of ischemic stroke, and the independent variables were ABPV and positive factors (*p* < 0.05) in univariate analysis, including age, sex, coronary heart disease, NIHSS, smoking, triglyceride, hyperhomocysteinemia, hemoglobin, serum creatinine, fasting blood glucose, and antihypertensive drugs. To reduce collinearity between the data, the blood pressure-dependent variables were not entered into the multivariate logistic regression model simultaneously. Data analysis was performed using IBM SPSS Statistics software version 26.0 (IBM SPSS Inc, Chicago, USA). The effect estimates were determined using 95% confidence intervals (CIs) with statistical significance defined as a *p*-value of < 0.05.

Random forest is a classic machine learning algorithm that is considered to have a high accuracy in disease risk prediction and diagnosis. The specific model construction process is as follows: (1) The random forest is assumed to have K-trees, and each tree has a certain number of sample sets to train. The sample set is randomly generated in proportion to the original training sample set of N, using the bootstrapping resampling method. (2) M is the number of features. For each tree node, m features are randomly selected, with m being much smaller than M. The Gini coefficient is used to compute the best splitting based on the m features. (3) In this study, 500 decision trees were constructed, and variables were randomly selected at each decision tree node. The optimal feature was selected from the m features, and the decision trees constituted the random forest. R software (version 3.5.1) was used for data analysis.

## 3. Results

### 3.1. Patient characteristics

**Figure 1:**
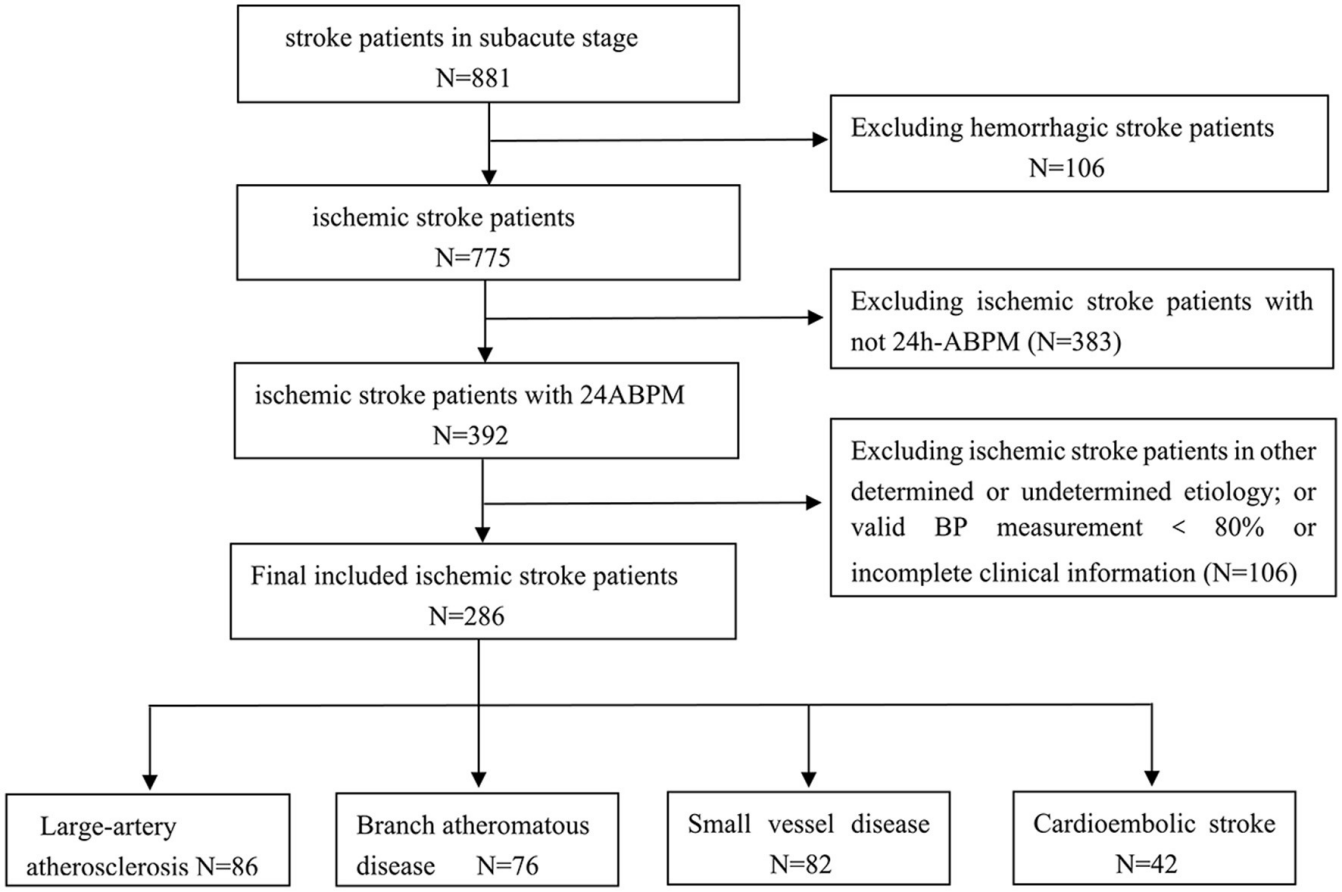
Flowchart of patients included in the study. ABPM, ambulatory blood pressure monitoring.

A total of 881 patients with stroke in the subacute stage were admitted to our hospital during the study period. Of these, 106 patients with hemorrhagic stroke were excluded, and an additional 383 patients who did not undergo the 24-h ABPM were also excluded. Ultimately, 286 patients were successfully enrolled in the final analysis after further excluding 106 patients with other determined/undetermined etiology infarcts or other reasons (Figure 1). Baseline characteristics and 24-h ambulatory blood pressure variability were compared across the ischemic stroke subtypes. The average age ranged from 47 to 95 years (mean ± SE: 75.3 ± 11.3), and approximately half of the patients (52.4%) were men. The patients were divided into four groups according to the etiological mechanism of ischemic stroke: 86 patients had large-artery atherosclerosis (30.1%), 76 had branch atheromatous disease (26.6%), 82 had small-vessel disease (28.7%) and 42 had cardioembolic strokes (14.7%).

A comparison of the clinical characteristics and laboratory variables is shown in Table 1. Among the four groups, there were significant differences in age (*p* < 0.001), sex (*p* = 0.005), smoking (*p* = 0.004), NIHSS (*p* < 0.001), TG (*p* = 0.005), FBG (*p* = 0.005), HCY (*p* = 0.041), Cr (*p* = 0.002), CHD (*p* = 0.012), HGB (*p* = 0.001), and β-blocker drugs (*p* < 0.001) among the four groups. No significant differences were found in alcohol intake, history of diabetes, hypertension, or levels of low-density lipoprotein, high-density lipoprotein, total cholesterol, C-reactive protein, uric acid, and antihypertensive drugs (Dihydropyridine/ARB/ACEI/Diuretics). Furthermore, multiple comparisons showed that there were significant differences in the positive factors in the univariate analysis between the four groups (Supplementary Figure 2).

**Table 1:** General characteristics of all subjects in the four ischemic stroke subtypes.

**Characteristics**	**LAA**	**BAD**	**SVD**	**CES**	**F/χ^2^/Z**	***P*-value**
	**(*****n** =* **86)**	**(*****n** =* **76)**	**(*****n** =* **82)**	**(*****n** =* **42)**		
Age, years^a^	74.1 ± 10.6	68.2 ± 11.3	80.5 ± 9.2	80.7 ± 8.8	23.596	< 0.001
Sex, male (%)	53 (61.6%)	40 (52.6%)	45 (54.9%)	12 (28.6%)	12.702	0.005
Smoking, yes (%)	27 (31.4%)	29 (38.2%)	13 (15.9%)	7 (16.7%)	13.194	0.004
Drinking, yes (%)	16 (18.6%)	14 (18.4%)	7 (8.5%)	6 (14.3%)	4.269	0.234
History of Diabetes mellitus (%)	41 (47.7%)	44 (57.9%)	38 (46.3%)	18 (42.9%)	3.322	0.345
History of hypertension (%)	69 (80.2%)	64 (84.2%)	65 (79.3%)	36 (85.7%)	1.225	0.747
History of CHD (%)	35 (40.7%)	20 (26.3%)	26 (31.7%)	23 (54.8%)	10.926	0.012
NIHSS^b^	8 (3, 11)	5 (2, 8)	0 (0, 4)	12 (5, 17)	67.110	< 0.001
low-density lipoprotein,^a^ mmol/L	2.2 ± 0.7	2.4 ± 0.9	2.2 ± 0.6	2.2 ± 0.7	1.561	0.199
high-density lipoprotein,^a, b^ mmol/L	0.96 (0.80, 1.14)	1.03 (0.83, 1.18)	1.02 (0.88, 1.25)	1.1 ± 0.4	3.336	0.343
Total cholesterol,^a^ mmol/L	3.7 ± 0.9	4.1 ± 1.2	3.9 ± 0.8	3.6 ± 1.0	2.261	0.082
Triglycerides,^b^ mmol/L	1.2 (0.9, 1.6)	1.4 (1.0, 1.8)	1.2 (0.9, 1.4)	1.1 (0.9, 1.3)	12.742	0.005
FBG,^b^ mmol/L	5.6 (4.8, 6.5)	6.5 (5.3, 9.3)	5.6 (4.9, 7.0)	6.24 (5.0, 7.5)	12.691	0.005
HCY,^b^ mmol/L	12.1 (9.4, 15.8)	10.7 (8.6, 12.9)	11.7 (9.8, 15.7)	11.2 (8.6, 14.3)	8.231	0.041
CRP,^b^mg/L	4.1 (1.2, 11.7)	2.3 (1.0, 9.4)	3.1 (1.2, 9.8)	3.7 (1.7, 14.3)	4.209	0.240
Creatinine,^b^ umol/L	70.5 (62.7, 87.0)	63.0 (52.3, 77.7)	71.5 (59.7, 90.0)	61.0 (46.7, 82.2)	15.070	0.002
Uric Acid,^a^ mmol/L	296.3 ± 82.4	297.1 ± 100.9	304.4 ± 103.4	267.6 ± 106.8	1.377	0.250
HGB,^a^ mg/L	127.1 ± 16.9	130.5 ± 16.9	123.1 ± 16.5	119.1 ± 15.5	5.300	0.001
**Class of antihypertensive drugs**						
Dihydropyridine CCB (%)	52 (60.5%)	44 (57.9%)	44 (53.7%)	20 (47.6%)	2.185	0.535
ARB (%)	35 (40.7%)	33 (43.4%)	30 (36.6%)	14 (33.3%)	1.491	0.684
ACEI (%)	8 (9.3%)	3 (3.9%)	1 (1.2%)	2 (4.8%)	6.116	0.106
β-Blocker (%)	16 (18.6%)	7 (9.2%)	13 (15.9%)	19 (45.2%)	23.826	< 0.001
Diuretics (%)	21 (24.4%)	17 (22.4%)	19 (23.2%)	14 (33.3%)	2.003	0.572

a Mean ± SD;

b median (interquartile range). CHD, coronary heart disease; NIHSS, National Institute of Health Stroke Scale; FBG, fasting glucose; HCY, hyperhomocysteinemia; CRP, C-reactive protein; HGB, hemoglobin; ACEI, angiotensin-converting enzyme inhibitor; ARB, angiotensin-receptor blocker; CCB, calcium-channel blocker; LAA: large-artery atherosclerosis; BAD: branch artery disease; SVD, small-vessel disease; CES, cardioembolic stroke.

### 3.2. Association between 24-h ABP levels and ischemic stroke subtypes

Table 2 presents 24-h ABP levels over the 24 h, daytime, and nighttime, with ischemic stroke subtypes. There were significant differences between mean SBP levels, SBP-SD, SBP-CV, mean DBP level, DBP-SD, and DBP-CV at 24 h, daytime and nighttime (*p* < 0.05). However, the statistically different was not in SBP-CV (*p* = 0.051) and DBP-CV (*p* = 0.200) among the four groups at nighttime. Furthermore, multiple comparisons among the four groups revealed that the BP and BPV during 24 h, daytime, and nighttime with ischemic stroke also have statistical differences (Supplementary Figure 2).

**Table 2:** Ambulatory blood pressure levels and variability in different subgroups.

**BP variate**	**LAA**	**BAD**	**SVD**	**CES**	**F/Z**	**P -value**
	**(*****n** =* **86)**	**(*****n** =* **76)**	**(*****n** =* **82)**	**(*****n** =* **42)**		
**24-hour**						
SBP^a^, mmHg	145.0 ± 16.8	140.5 ± 14.3	138.0 ± 15.9	132.4 ± 16.9	6.473	< 0.001
DBP^a^,mmHg	78.2 ± 10.7	79.3 ± 10.7	72.5 ± 10.9	73.9 ± 10.5	6.912	< 0.001
SBP SD^b^, mmHg	15.9 (13.4, 19.4)	13.2 (11.0, 16.3)	14.1 (11.9, 17.5)	16.5 (13.5, 18.2)	6.514	< 0.001
DBP SD^a, b^, mmHg	10.1 (8.2, 12.1)	9.6 ± 2.7	9.3 (7.6, 11.3)	11.0 (8.9, 13.1)	5.140	0.011
SBP CV^b^, %	11.2 (9.3, 13.0)	9.7 (7.7, 11.6)	10.7 ± 2.9	12.6 ± 3.1	8.608	< 0.001
DBP CV^a, b^, %	12.9 (10.8, 15.3)	12.2 ± 3.6	13.3 (10.7, 15.5)	15.2 (12.0, 18.8)	7.248	0.002
**Daytime**						
SBP^a^, mmHg	144.8 ± 17.7	140.9 ± 14.3	138.0 ± 16.3	131.7 ± 17.6	6.449	< 0.001
DBP^a^, mmHg	78.0 ± 11.1	79.8 ± 10.8	73.4 ± 12.1	73.5 ± 10.9	5.615	0.001
SBP SD^b^, mmHg	15.3 (12.3, 17.9)	13.4 (10.8, 15.9)	14.4 (11.7, 17.6)	15.8 (12.3, 17.6)	4.320	0.003
DBP SD^a, b^, mmHg	9.8 (7.7, 11.6)	9.2 ± 2.7	9.1 (7.6, 11.6)	10.3 (8.6, 12.1)	3.678	0.047
SBP CV^a, b^, %	10.7 (8.7, 12.0)	9.6 ± 2.6	10.6 (8.8, 12.4)	12.2 ± 2.9	7.363	< 0.001
DBP CV^a, b^, %	12.6 (10.4, 15.1)	11.7 ± 3.5	13.0 (10.3, 16.0)	14.3 (12.0, 17.0)	7.200	0.001
**Nighttime**						
SBP^a^, mmHg	144.2 ± 18.4	137.3 ± 16.7	138.4 ± 16.8	134.9 ± 17.6	3.530	0.015
DBP^a^, mmHg	78.2 ± 11.2	75.8 ± 11.6	70.9 ± 12.2	76.4 ± 11.1	6.068	0.001
SBP SD^a, b^, mmHg	14.2 (10.7, 23.0)	11.5 (8.9, 14.4)	12.2 ± 4.9	13.5 (9.6, 16.9)	4.063	0.018
DBP SD^b^, mmHg	8.8 (7.1, 11.3)	7.9 (6.1, 10.0)	8.0 (6.1, 9.4)	9.0 (6.8, 11.9)	4.825	0.026
SBP CV^a, b^, %	9.8 (7.4, 11.7)	8.3 (6.1, 11.0)	8.8 ± 3.2	10.0 (6.7, 13.2)	2.640	0.051
DBP CV^a, b^, %	11.4 (9.2, 15.4)	10.6 (8,2, 13.4)	11.6 ± 4.3	12.5 (9.4, 15.9)	2.398	0.200
**Nocturnal dip**					19.926	0.018
Reverse dipper (< 0%)	38 (44.2%)	26 (34.2%)	47 (57.3%)	27 (64.3%)		
Non-dipper (0–9%)	38 (44.2%)	35 (46.1%)	28 (34.1%)	12 (28.6%)		
Dipper (10–19%)	7 (8.1%)	14 (18.4%)	7 (8.5%)	2 (4.8%)		
Extreme dipper (≥20%)	3 (3.5%)	1 (1.3%)	0 (0)	1 (2.4%)		

a Mean ± SD;

b median (interquartile range). SBP, systolic blood pressure; DBP, diastolic blood pressure; CV, coefficient of variation; SD, standard deviation. LAA, large-artery atherosclerosis; BAD, branch artery disease; SVD, small-vessel disease; CES, cardioembolic stroke.

### 3.3. Feature selection and ranking by random forest

**Figure 2:**
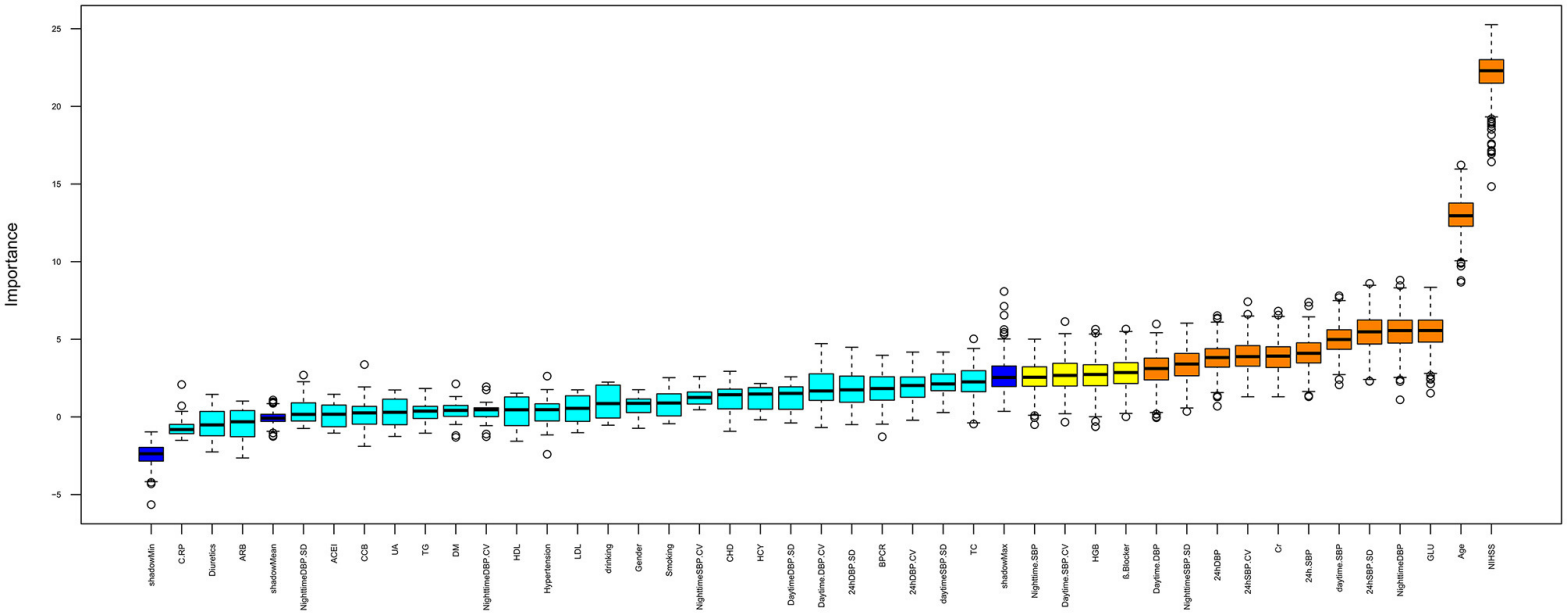
Important feature selection by random forest. The plot displays all the features leading up to the stroke. Important variables are in orange boxes, rejected variables are in cyan boxes, and tentative variables are in yellow boxes.

Figure 2 displays the important variables that contribute to the risk of ischemic stroke as identified by random forest: NIHSS, age, GLU, Cr, BP, and BPV (24-h SBP, 24-h SBP-SD, 24-h SBP-CV, 24-h DBP, daytime SBP, daytime DBP, nighttime DBP, and nighttime SBP-SD). These results support the notion that SB and BPV are stronger risk factors for ischemic stroke. To further analyze the difference between BP and BPV in ischemic stroke subtypes, multinomial logistic regression was conducted.

### 3.4. The multinomial logistic regression analysis association between 24-h ABP levels and stroke subtypes

Table 3 shows the multinomial logistic regression analysis between 24-h blood pressure variability and stroke subtypes. The results indicated that a higher SBP is an independent risk factor for LAA over 24 h, daytime and nighttime, compared with other stroke subtypes after adjusting for age, sex, CHD , β-blocker drugs, smoking, NIHSS, TG, FBG, HCY, Cr, and HGB (*p* < 0.05). Higher SBP-SD and SBP-CV were also associated with an increased risk of LAA stroke compared to BAD and SVD groups (*p* < 0.05). Meanwhile, when compared with the BAD and SVD groups, the nighttime mean DBP level and DBP-SD were significantly associated with greater risk in the CES group (*p* < 0.05). This indicates that the DBP variability at night was an independent risk factor for CES.

**Table 3:** Multinomial logistic regression analysis: association between 24-h ABP levels and stroke subtypes.

	**Model 1**				**Model 2**			**Model 3**	
	**CES**	**LAA**	**BAD**	**SVD**	**SVD**	**LAA**	**BAD**	**BAD**	**LAA**
	**ref**	**OR (95%CI)**	**OR (95%CI)**	**OR (95%CI)**	**ref**	**OR (95%CI)**	**OR (95%CI)**	**ref**	**OR (95%CI)**
**24-hour**									
SBP, mmHg	1	1.610 (1.192~2.173)^b^	1.221 (0.880~1.694)	1.218 (0.892~1.663)	1	1.321 (1.053~1.657)^a^	1.002 (0.778~1.292)	1	1.318 (1.042~1.688)^a^
DBP, mmHg	1	0.926 (0.600~1.430)	0.819 (0.502~1.337)	0.675 (0.420~1.084)	1	1.372 (0.953~1.974)	1.214 (0.811~1.817)	1	1.131 (0.788~1.622)
SD _SBP_, mmHg	1	1.166 (0.767~1.772)	0.709 (0.425~1.183)	0.779 (0.484~1.252)	1	1.498 (1.018~2.203)^a^	0.911 (0.578~1.436)	1	1.644 (1.086~2.489)^a^
SD _DBP_, mmHg	1	0.753 (0.422~1.343)	0.693 (0.351~1.366)	0.454 (0.236~0.873)^a^	1	1.661 (0.973~2.834)	1.527 (0.832~2.802)	1	1.087 (1.626~1.890)
SBP-CV, %	1	1.078 (0.569~1.075)	0.516 (0.238~1.116)	0.754 (0.375~1.514)	1	1.426 (0.814~2.498)	0.684 (0.349~1.340)	1	2.085 (1.117~3.890)^a^
DBP-CV, %	1	0.696 (0.424~1.142)	0.556 (0.308~1.002)	0.582 (0.336~1.008)	1	1.196 (0.758~1.888)	0.956 (0.565~1.617)	1	1.252 (0.774~2.024)
**Daytime**									
SBP, mmHg	1	1.641 (1.229~2.191)^b^	1.360 (0.994~1.863)	1.260 (0.934~1.700)	1	1.302 (1.045~1.623)^a^	1.080 (0.846~1.377)	1	1.206 (0.965~1.507)
DBP, mmHg	1	0.930 (0.635~1.362)	0.808 (0.522~1.252)	0.766 (0.511~1.147)	1	1.215 (0.877~1.683)	1.055 (0.728~1.530)	1	1.151 (0.816~1.626)
SD _SBP_, mmHg	1	1.186 (0.745~1.888)	0.757 (0.433~1.324)	0.960 (0.575~1.602)	1	1.236 (0.823~1.855)	0.789 (0.486~1.279)	1	1.566 (1.002~2.447)^a^
SD _DBP_, mmHg	1	0.739 (0.395~1.383)	0.692 (0.337~1.417)	0.541 (0.274~1.068)	1	1.367 (0.804~2.323)	1.278 (0.701~2.331)	1	1.069 (0.613~1.864)
CV _SBP_, %	1	0.879 (0.475~1.628)	0.559 (0.271~1.153)	0.694 (0.352~1.370)	1	1.266 (0.728~2.203)	0.805 (0.428~1.515)	1	1.573 (0.875~2.826)
CV _DBP_, %	1	0.829 (0.519~1.324)	0.699 (0.401~1.219)	0.812 (0.488~1.352)	1	1.020 (0.660~1.578)	0.861 (0.520~1.425)	1	1.186 (0.744~1.889)
**Nighttime**									
SBP, mmHg	1	1.315 (1.022~1.692)^a^	1.064 (0.800~1.416)	1.126 (0.862~1.470)	1	1.168 (0.954~1.431)	0.946 (0.748~1.196)	1	1.235 (0.995 ~1.534)
DBP, mmHg	1	0.873 (0.596~1.280)	0.609 (0.393~0.944)^a^	0.592 (0.394~0.891)^a^	1	1.475 (1.071~2.033)^a^	1.029 (0.720~1.469)	1	1.434 (1.021~2.015)^a^
SD _SBP_, mmHg	1	1.202 (0.862~1.678)	0.838 (0.559~1.256)	0.864 (0.595~1.255)	1	1.391 (1.033~1.874)^a^	0.969 (0.680~1.381)	1	1.435 (1.049~1.965)^a^
SD _DBP_, mmHg	1	0.688 (0.438~1.079)	0.469 (0.268~0.821)^b^	0.487 (0.278~0.852)^a^	1	1.413 (0.886~2.253)	0.964 (0.569~1.631)	1	1.466 (0.934~2.300)
CV _SBP_, %	1	1.098 (0.711~1.696)	0.753 (0.452~1.255)	0.702 (0.428~1.149)	1	1.565 (1.051~2.329)^a^	1.073 (0.682~1.689)	1	1.458 (0.984~2.160)
CV _DBP_, %	1	0.974 (0.905~1.048)	0.929 (0.851~1.014)	0.929 (0.852~1.013)	1	1.316 (0.945~1.833)	1.026 (0.703~1.498)	1	1.282 (0.917~1.794)

Model 1: CES is used as a reference; the multinomial logistic regression model was adjusted for age, sex, NIHSS, coronary heart disease, smoking, triglyceride, hyperhomocysteinemia, hemoglobin, serum creatinine, fasting blood glucose, and antihypertensive drugs plus 24-h BP plus BPV. Model 2: SVD is used as a reference, and Model 3 BAD is used as a reference, respectively, and the multivariate regression model includes the same analysis variables as Model 1. The results of the multinomial logistic regression analysis were presented as OR per 10 mmHg increase in BP, 5 mmHg increase in SD, and 5% increase in CV.

a p < 0.05,

b p < 0.01. OR, odds ratio; CI, confidence interval; LAA, large-artery atherosclerosis; BAD, branch artery disease; SVD, small-vessel disease; CES, cardioembolic stroke.

## 4. Discussion

In this study, we explored the potential association between 24-h ABP levels, 24-h ABPV, and subtypes of ischemic stroke in patients treated at our hospital. We discovered that higher SBP levels significantly increased the risk of large-artery atherosclerosis stroke at 24 h, daytime, and nighttime. Multinomial logistic regression analysis indicated that the SBP level of 24 h was an independent risk factor for large-artery atherosclerosis after adjusting for patient demographics and vascular risk factors. When comparing the risks of SVD and BAD to LAA, SBP-SD, SBP-CV, and DBP levels, these were significantly associated with large-artery atherosclerosis, especially at night. At the same time, nighttime DBP levels and DBP-SD were independent risk factors for CES compared to BAD and SVD, indicating that a higher nighttime diastolic blood pressure variability (DBPV) significantly increased the risk of CES.

We believe that higher SBP levels are strongly associated with ischemic stroke and are a stronger independent risk factor for the strict control and management of hypertension (26). A high mean SBP level and high SBPV were significantly associated with poor functional outcomes of acute ischemic stroke and/or may increase the risk of recurrent stroke during long-term secondary stroke prevention (26–28). This suggests that effective control of the SBPV is important for reducing stroke events. Nonetheless, very few studies have further analyzed the characteristics of blood pressure levels and blood pressure variability in subtypes of ischemic stroke, which may underlie different pathogeneses (29, 30).

The present study showed significant differences in mean BP and BPV between the four ischemic stroke subtypes. First, the SBP in the LAA group was higher than that in the CES, BAD, and SVD groups, similar to previous studies on the contribution of hypertension to atherosclerosis. It has been reported that angiotensin II, a key factor in the pathogenesis of hypertension, and T cells, the main biomarkers of inflammation, simultaneously take part in the unleashing of inflammatory pathways causing elevated blood pressure and atherosclerosis. These pathophysiological changes can lead to an increase in the intima-media thickness of large vessels and the formation of plaque in the artery leading to atherosclerosis; furthermore, the large vessel is gradually stenosed or occluded in the subsequent stages (31, 32).

When compared with BPV among the four groups, we found that SBP-SD (24 h, daytime, and nighttime) was higher in the LAA group compared to the BAD group. Additionally, the SPB-CV at 24 h and the nighttime DBP level showed similar results. Branch atheromatous disease, mainly involving the lenticulostriate arterial and the pontine penetrating arterial, with a smaller blood vessel diameter than the parental artery, was considered to have the same risk factors (hypertension, diabetes mellitus, hyperlipidemia, and hyperhomocysteinemia) as a large atherosclerotic disease. However, the etiology may differ for small and large cerebral arterial diseases. The present study shows that SBPV is higher in LAA than that in BAD, which supports a strong correlation between SBPV with large-artery atherosclerosis stroke (27). Moreover, the clinical characteristics also differed between the LAA and BAD groups. Patients in the LAA group were significantly older than those in the BAD group. Therefore, BAD may be considered an early stage of large-artery atherosclerosis and should be strictly managed with SBP and SBPV. Meanwhile, multinomial logistic regression analysis depicted that the fasting blood glucose (FBG) level was an independent risk factor for BAD. These results are consistent with previous studies showing that DM or HbA1c is associated with BAD, especially paramedian pontine arteries disease (33).

The SBP-SD in the LAA group (24 h, nighttime) was higher than that in the SVD groups, and the same significance was in the mean DBP level and SBP-CV at nighttime. Previous studies have demonstrated that large SBP variability is associated with a higher burden of cerebral small-vessel disease (13, 34). Our research found a difference in SBP variability between LAA and SVD, which may be attributed to their different etiologies. Small-vessel disease, in particular, is caused by a group of pathological processes of the perforating cerebral arterioles, capillaries, and venules of the brain and is considered lipohyalinosis of the penetrating artery. LAA is caused by large-artery atherosclerosis with stenosis or blocking. The apparent differences in anatomy and pathophysiology may explain the differences in the SBP variability.

When comparing BP levels and BPV with BAD and SVD in the CES group, the results demonstrated that nighttime DBP levels and DBP-SD are independent risk factors for CES. In addition, elevated DBP levels and DBPV increase the risk of CES events at night. This is consistent with a multicenter, prospective cohort study, which suggested that a higher nighttime BP and a rising pattern of nocturnal BP were significantly associated with the risk of cardiovascular disease in patients who underwent 24-h ambulatory BP monitoring. Furthermore, nighttime BP was more important than daytime BP as a risk factor for total cardiovascular disease burden (15). There are three possible explanations for this finding. First, higher blood pressure variability notably increased the risk of atrial fibrillation (AF) (35). Being an independent risk factor for ischemic stroke, it increased the incidence of stroke four to five times compared to the patients without atrial fibrillation (36). The second was a heart rate, which was higher in the CES group than in the BAD and SVD groups at night during the 24-h ambulatory BP monitoring period. These are often accompanied by atrial fibrillation or other cardiovascular diseases. Increased and irregular heart rates shorten the duration of diastole in the ventricles, resulting in higher diastolic blood pressure. Third, the changes and characteristics of hypertension in older patients, the reduction in vascular elasticity, and baroreceptor reflex dysfunction increase the fluctuation of blood pressure and the impairment of autoregulation of blood flow in vital organs (37). In the current study, patients in the BAD group were younger than those in the CES group. All of these factors contribute to the development of CES by increasing DBP levels and DBP-SD at night.

The current study had some limitations: First, the classification of subtypes of ischemic stroke based on brain vascular images was not performed with a high-resolution MRI, which is useful in identifying atherosclerotic pathologies in perforated arteries, providing direct evidence to distinguish between LAA and BAD. Second, our study was based on a single-center retrospective analysis, and the limited data may have affected the statistical validity of BP, BPV, and ischemic stroke subtypes. We believe that multicenter, prospective studies are needed to obtain more significant results on this issue. Third, patients underwent a 24-h ABPM while in the hospital, which may have led to some degree of white coat hypertension. A larger and prospective study is needed to further explore the differences in 24-h ABPM measurements between inpatient and outpatient settings for ischemic stroke patients.

## 5. Conclusion

There were significant differences in BP and BPV among the ischemic stroke subtypes in the subacute stage. Higher systolic blood pressure and systolic blood pressure variability during the 24 h, daytime, and nighttime, and nighttime diastolic blood pressure were independent predictors of large-artery atherosclerosis stroke. Increased nighttime diastolic blood pressure variability was found to be an independent risk factor for cardioembolic stroke.

## Data availability statement

The original contributions presented in the study are included in the article/Supplementary material, further inquiries can be directed to the corresponding author.

## Ethics statement

The studies involving human participants were reviewed and approved by the Ethics Committe of Beijing Zhongguancun Hospital. Written informed consent for participation was not required for this study in accordance with the national legislation and the institutional requirements.

## Author contributions

XLi contributed significantly to the conception of the study and modified the manuscript. LijW accumulated clinical data, discussed results, and contributed to the final version of the manuscript. XX, ZC, and PL modified the manuscript. XLiu contributed to the imaging analysis. GW, DY, and YW have provided statistical analysis support. LinW provided constructive and forward-looking advice during the research. All authors contributed to the article and approved the submitted version.

## Supplementary material

Supplementary Figure 1 Multiple comparisons in demographic information and clinical characteristics. Univariate analysis found that sex, smoking, coronary heart disease, β-blockers, age, hemoglobin, NIHSS, FBG, HCY, TG, and CR have positive differences between groups. Multiple comparison analyses revealed significant differences within groups. ^*^*p* < 0.05, ^**^*p* < 0.010, and ^***^*p* < 0.001.

Supplementary Figure 2 Multiple comparisons in 24-h ambulatory blood pressure. Multiple comparison analyses showed within-group differences in blood pressure levels and blood pressure variability. ^*^*p* < 0.05, ^**^*p* < 0.010, and ^***^*p* < 0.001.
