# Supplementary figures and images for: Twenty-four-hour ambulatory blood pressure variability and association with ischemic stroke subtypes in the subacute stage
Source: Front Neurol. 2023 Apr 17;14:1139816. doi: 10.3389/fneur.2023.1139816 (PMC10149864; doi:10.3389/fneur.2023.1139816)

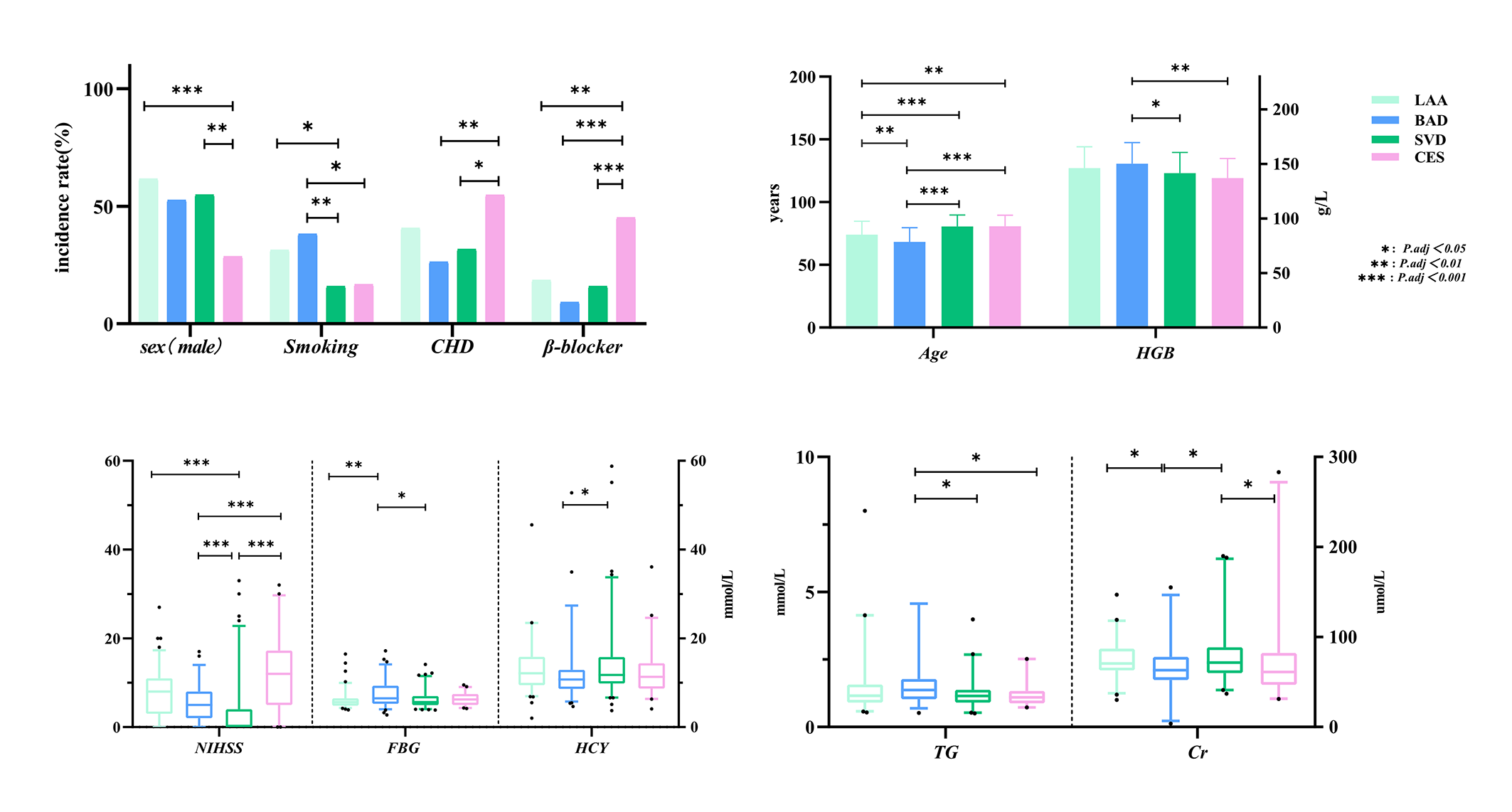

Supplement: Supplementary Figure 1 — Multiple comparisons in demographic information and clinical characteristics. Univariate analysis found that sex, smoking, coronary heart disease, β-blockers, age, hemoglobin, NIHSS, FBG, HCY, TG, and CR have positive differences between groups. Multiple comparison analyses revealed significant differences within groups. *p < 0.05, **p < 0.010, and ***p < 0.001. [file Image_1.TIF]

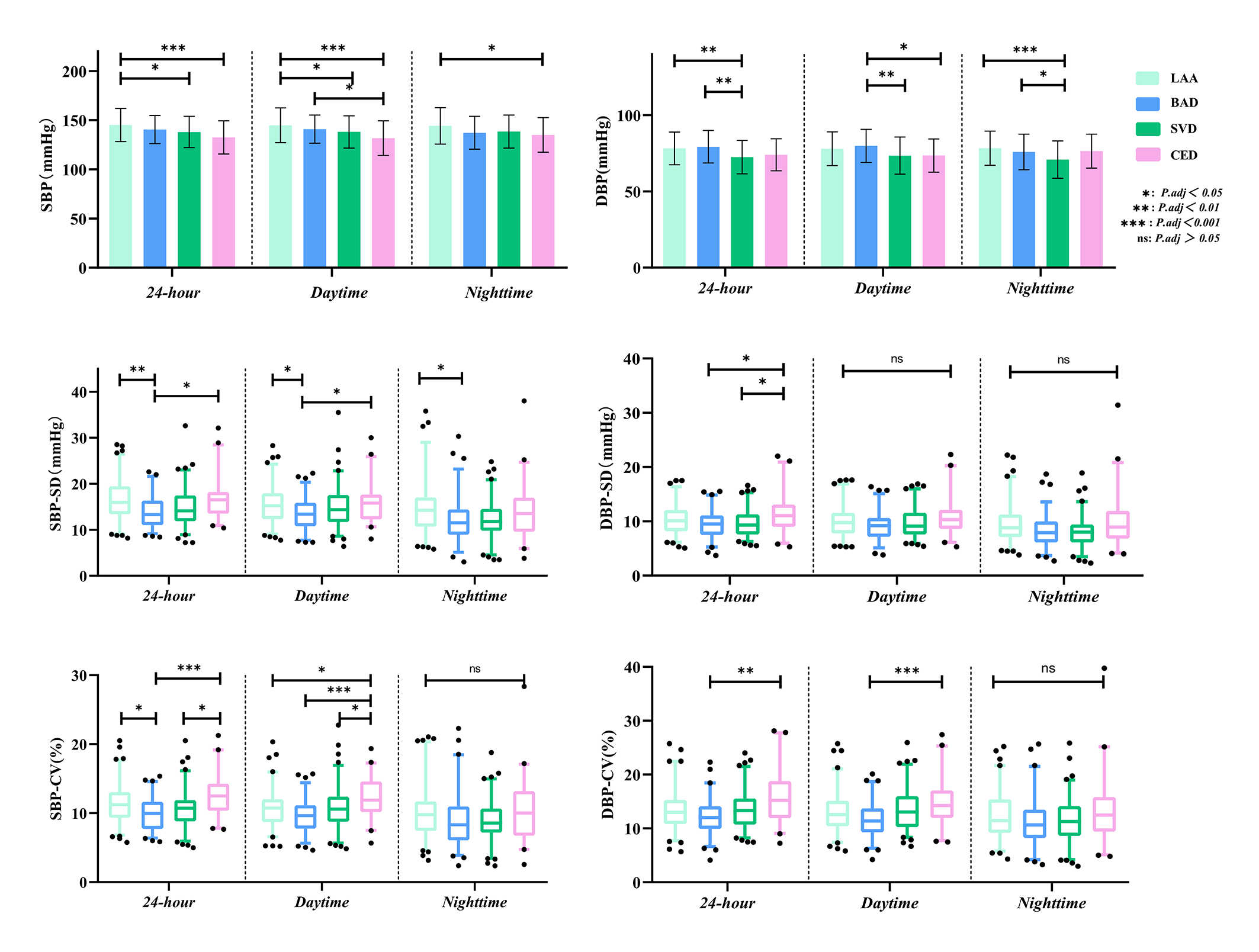

Supplement: Supplementary Figure 2 — Multiple comparisons in 24-h ambulatory blood pressure. Multiple comparison analyses showed within-group differences in blood pressure levels and blood pressure variability. *p < 0.05, **p < 0.010, and ***p < 0.001. [file Image_2.TIF]
